# Coassembly and binning of a twenty-year metagenomic time-series from Lake Mendota

**DOI:** 10.1038/s41597-024-03826-8

**Published:** 2024-09-04

**Authors:** Tiffany Oliver, Neha Varghese, Simon Roux, Frederik Schulz, Marcel Huntemann, Alicia Clum, Brian Foster, Bryce Foster, Robert Riley, Kurt LaButti, Robert Egan, Patrick Hajek, Supratim Mukherjee, Galina Ovchinnikova, T. B. K. Reddy, Sara Calhoun, Richard D. Hayes, Robin R. Rohwer, Zhichao Zhou, Chris Daum, Alex Copeland, I-Min A. Chen, Natalia N. Ivanova, Nikos C. Kyrpides, Nigel J. Mouncey, Tijana Glavina del Rio, Igor V. Grigoriev, Steven Hofmeyr, Leonid Oliker, Katherine Yelick, Karthik Anantharaman, Katherine D. McMahon, Tanja Woyke, Emiley A. Eloe-Fadrosh

**Affiliations:** 1 DOE Joint Genome Institute, Lawrence Berkeley National Laboratory, Berkeley, CA 94720 USA; 2 Department of Biology, Spelman College, Atlanta, GA 30314 USA; 3 Environmental Genomics and Systems Biology Division, Lawrence Berkeley National Laboratory, Berkeley, CA 94720 USA; 4 Department of Bacteriology, University of Wisconsin-Madison, Madison, WI 53706 USA; 5 Department of Plant and Microbial Biology, University of California Berkeley, Berkeley, CA 94720 USA; 6 Applied Math and Computational Research Division, Lawrence Berkeley National Laboratory, Berkeley, CA 94720 USA; 7 Electrical Engineering and Computer Sciences Department, University of California Berkeley, Berkeley, CA 94720 USA; 8 Department of Civil and Environmental Engineering, University of Wisconsin-Madison, Madison, WI 53706 USA; 9 Life and Environmental Sciences, University of California Merced, Merced, CA 95343 USA; 10 Present Address: Department of Integrative Biology, The University of Texas at Austin, Austin, TX 78712 USA

**Keywords:** Data processing, Microbial ecology

## Abstract

The North Temperate Lakes Long-Term Ecological Research (NTL-LTER) program has been extensively used to improve understanding of how aquatic ecosystems respond to environmental stressors, climate fluctuations, and human activities. Here, we report on the metagenomes of samples collected between 2000 and 2019 from Lake Mendota, a freshwater eutrophic lake within the NTL-LTER site. We utilized the distributed metagenome assembler MetaHipMer to coassemble over 10 terabases (Tbp) of data from 471 individual Illumina-sequenced metagenomes. A total of 95,523,664 contigs were assembled and binned to generate 1,894 non-redundant metagenome-assembled genomes (MAGs) with ≥50% completeness and ≤10% contamination. Phylogenomic analysis revealed that the MAGs were nearly exclusively bacterial, dominated by Pseudomonadota (Proteobacteria, N = 623) and Bacteroidota (N = 321). Nine eukaryotic MAGs were identified by eukCC with six assigned to the phylum Chlorophyta. Additionally, 6,350 high-quality viral sequences were identified by geNomad with the majority classified in the phylum Uroviricota. This expansive coassembled metagenomic dataset provides an unprecedented foundation to advance understanding of microbial communities in freshwater ecosystems and explore temporal ecosystem dynamics.

## Background & Summary

The North Temperate Lakes Long-Term Ecological Research (NTL-LTER) program^1^ plays a vital role in advancing ecological science by providing long-term, in-depth data and insights into the complex dynamics of freshwater ecosystems. The extensive data collected by NTL-LTER not only aids in unraveling the intricate relationships between species and their environment, but also informs broader ecological research and policy decisions, making it an indispensable resource for the scientific community. The primary NTL-LTER study sites include a set of seven northern Wisconsin and four southern Wisconsin lakes and their surrounding landscapes.

**Fig. 1:**
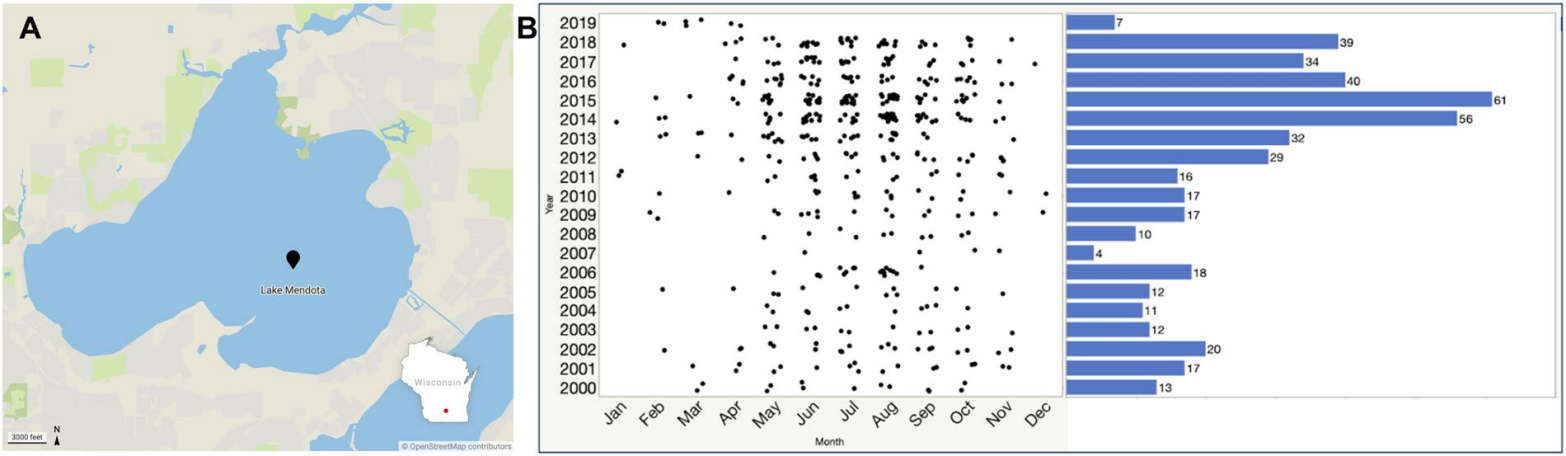
Lake Mendota sample collection. (**A**) Lake Mendota is located in Madison, Wisconsin, as indicated by the red dot in the lower right inset. All samples part of this study were collected from the NTL-LTER site located at the center of Lake Mendota (latitude = 43.0995, longitude = −89.4045). (**B**) Time-series of the 471 samples collected from Lake Mendota between 2000 – 2019. Sampling time points are indicated by black dots by month (x-axis) and year (y-axis), while the total number of samples collected per year is indicated by the horizontal bar plots.

**Fig. 2:**
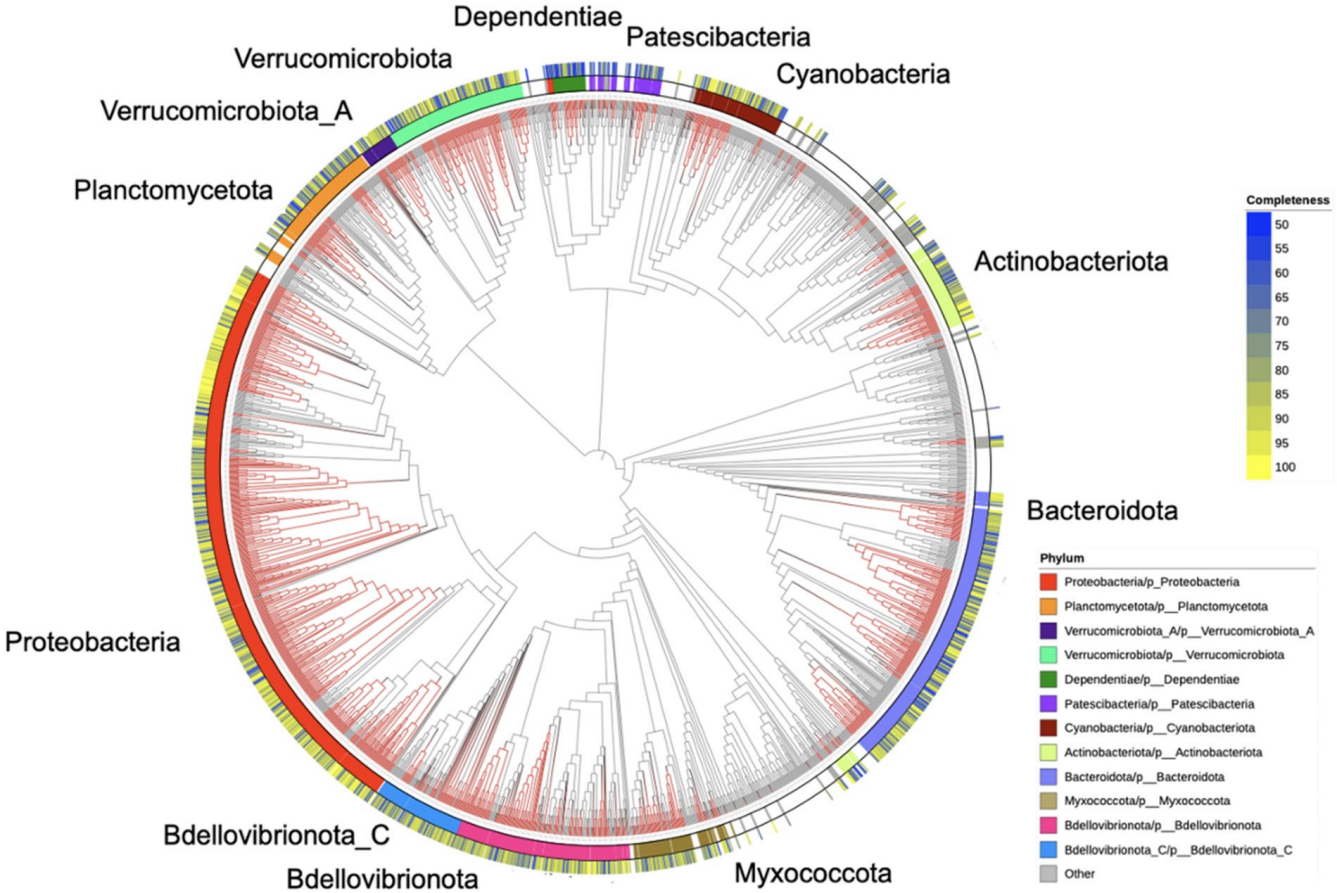
Phylogenetic tree of the bacterial MAGs. Concentric rings moving outward from the tree show the inferred phylum-level taxonomy and estimated level of genome completeness. Red branches indicate MAGs from the coassembly and branches in black represent family-level representative genomes from the GTDB database (release 95). Phyla are named based on IMG/M taxonomic assignment followed by phylogenetic affiliation according to the Genome Taxonomy Database (GTDB) release 95. Branch lengths are shown simplified and not to true scale.

**Fig. 3:**
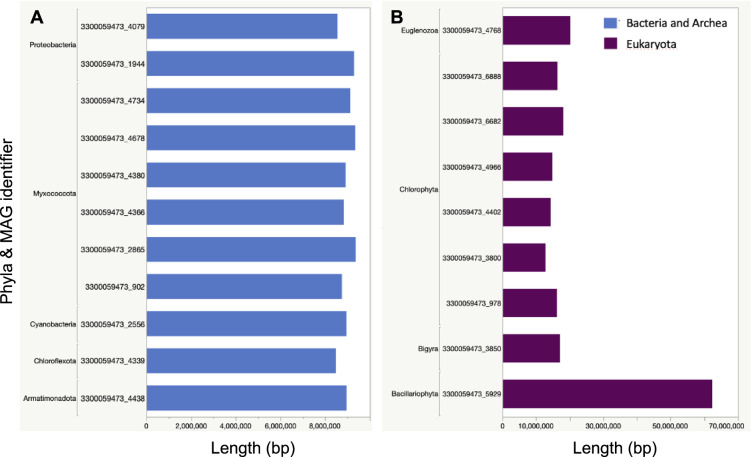
Phylum-level taxonomy and assembly size of the twenty largest MAGs. MAGs are separated by (**A**) prokaryote and (**B**) eukaryote taxonomic affiliations.

Lake Mendota is a freshwater, eutrophic lake located in Madison, Wisconsin (Fig. 1a), and serves as one of several study sites serviced by the NTL-LTER program. In this study, we leveraged samples collected from the surface water of Lake Mendota between 2000 and 2019 (Fig. 1), primarily during ice-free periods^2^, to generate 471 shotgun metagenomes (PRJNA1056043)^3^. To maximize assembly and recovery of population genomes, all reads were coassembled (PRJNA1134257)^4^ using the distributed metagenome assembler MetaHipMer, which is the only metagenome assembler capable of handling terabase-scale datasets^5^. In comparison to multi-assembly methods, where samples are individually assembled and then contigs are combined, coassembly using MetaHipMer yields improved reconstruction of population genomes. In total, 95,523,664 contigs longer than 500 base pairs were generated and annotated using the DOE-JGI metagenome workflow (v5.1.11)^6^. MetaBAT2 (v2.15)^7^ binning yielded a total of 1,885 non-redundant bacterial and archeal metagenome-assembled genomes (MAGs) of medium- and high-quality with a CheckM^8^ (v1.1.3) estimated completeness of ≥50% and contamination of ≤10% (Table 1, Fig. 2). Phylogenomic analysis using GTDB-Tk, which is a software toolkit that assigns bacterial and archeal taxonomy based on the Genome Taxonomy Database (GTDB) (v1.3.0, GTDB database release 95)^9^, indicated that a majority of these MAGs belonged to the two phyla Pseudomonadota (Proteobacteria, N = 623) and Bacteroidota (N = 321) (Table 2). Additionally, nine eukaryotic MAGs were detected with six taxonomically affiliated with the class Trebouxiophyceae in the phylum Chlorophyta (Table 2 and Table S2). Four of these high-quality Trebouxiophyceae MAGs were further annotated using JGI’s PhycoCosm annotation pipeline^10^. The largest eukaryotic MAG was assigned to the phylum Bacillariophyta (bin ID: 3300059473_5929) and was approximately 62.3 Mb long (Fig. 3).

**Table 1:** Overview of Lake Mendota Coassembly Data.

Number of Metagenomes	471
Number of Contigs	95,523,664
Number of COG Clusters	4,631
Number of Pfam Clusters	14,961
Number of MetaBat Bins	1,885
Number of Eukaryotic MAGs	9

**Table 2:** Phylum-level taxonomic distribution of prokaryotic and eukaryotic MAGs.

Phylum	Total Count
**Bacteria**	
Pseudomonadota (Proteobacteria)	623
Bacteroidota	321
Bdellovibrionota	161
Verrucomicrobiota	132
Planctomycetota	115
Actinobacteriota	92
Cyanobacteria	86
Bdellovibrionota_C	82
Myxococcota	81
Patescibacteria	38
Verrucomicrobiota_A	29
Dependentiae	28
Chloroflexota	16
Acidobacteriota	13
Gemmatimonadota	12
Firmicutes	11
Other Bacteria	45
**Eukaryota**	
Chlorophyta	6
Bacillariophyta	1
Bigyra	1
Euglenozoa	1
Total	1,894

For bacterial phyla, only taxa with >10 bins are shown. The full list is available in Supplementary Table S1.

**Fig. 4:**
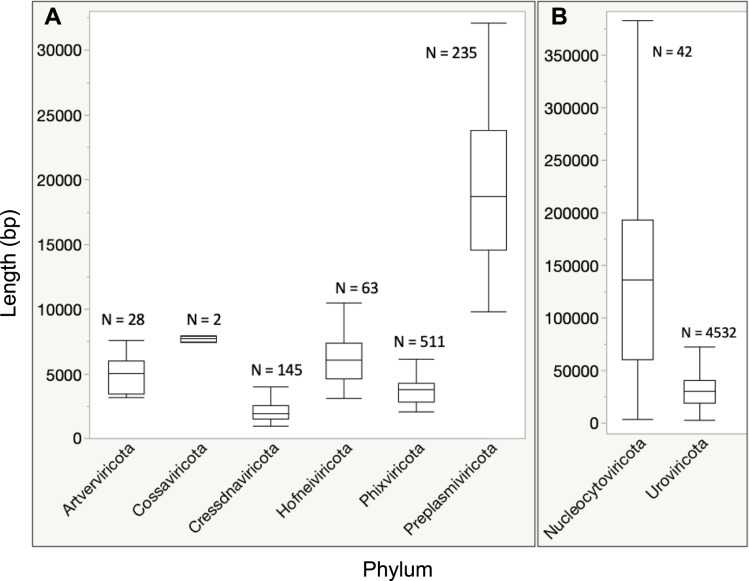
Viral genome size distribution. Viruses were taxonomically classified at the phylum level and total length per phyla is shown for genome length less than 20,000 kb (**A**) and genome length greater than 20,000 kb (**B**).

To complement the reconstruction of prokaryotic and eukaryotic MAGs, we next identified putative viral contigs and taxonomically classified them using geNomad (v1.7.4)^11^. We note that geNomad takes a conservative approach to avoid false positives compared to other viral identification tools, and thus might miss authentic viral contigs. CheckV (v1.5)^12^ was used to assess estimated completeness (AAI-based, medium or high confidence) of ≥50%, and excluding contigs longer than 150% of the aai_expected_length. A total of 6,530 unique viral sequences across 8 known viral phyla were identified (Table 3, Fig. 4). Viruses of the phylum Uroviricota represented 71.3% of viral sequences detected (N = 4,532). In addition, no completeness estimation could be obtained for another 26,625 predicted viral contigs ≥10 kb, some potentially representing large fragments of novel virus genomes. Data for all non-redundant MAGs and viral contigs are available under taxon identifier 3300059473 in JGI’s IMG/M platform^13^. This comprehensive dataset serves as a valuable resource for gaining insights into the dynamics of microbial and viral communities within freshwater ecosystems.

**Table 3:** Predicted virus contigs identified.

Phylum	Total Count
Uroviricota	4,532
Phixviricota	511
Preplasmiviricota	235
Cressdnaviricota	145
Hofneiviricota	63
Nucleocytoviricota	42
Artverviricota	28
Cossaviricota	2
Unknown	792
Total	6,350

## Methods

### Sample collection and DNA extraction

Samples collected from Lake Mendota were obtained through the NTL-LTER program (https://lter.limnology.wisc.edu/). Sample collection and DNA extraction, but not shotgun metagenome sequencing (described below), was completed as previously described by Rohwer and McMahon^2^. Briefly, surface layer (integrated 12 m epilimnion) water samples collected from the deepest location of Lake Mendota were filtered onto 0.2-μm pore-size polyethersulfone Supor filters (Pall Corp., Port Washington, NY, USA) prior to storage at −80 °C, allowing the collection of DNA from prokaryotic, eukaryotic, and viral species present in the sample. DNA was purified from these filters using FastDNA Spin Kits (MP Biomedicals, Burlingame, CA, USA). Detailed metadata is available through JGI’s Genomes OnLine (GOLD)^14^ system under GOLD Study ID Gs0136121.

### Sequencing, read QC, and filtering

For this study, standard True-Seq Illumina libraries were generated at the DOE Joint Genome Institute (JGI) and sequenced using the NovaSeq 6000 with the S4 flow cell. Data generation spanned a period of ~2.5 years, and thus software tool versions and protocols for read quality control and filtering differ slightly for each of the individual metagenomes. Further details can be found in Supplementary Dataset 1 which is organized by JGI sequencing project identifier. In general, BBDuk^13^ was used to remove contaminants, trim reads that contained adapter sequence, and right quality trim reads where quality drops to 0. BBDuk was used to remove reads that contained 4 or more ‘N’ bases, had an average quality score across the read less than 3 or had a minimum length < = 51 bp or 33% of the full read length. Reads mapped with BBMap^15^ to masked human, cat, dog, mouse, and common microbial contaminant references at 93% identity were separated into chaff files and discarded. The final filtered FASTQ was subsequently used for metagenome coassembly and mapping.

Filtered reads were coassembled with MetaHipMer^5^ v2.1.0.1.256-g6a25b79-dirty RevertAggrShuffleReads [mhm2.py -v–pin = none–checkpoint = true] on 1,500 nodes on the Summit system at the Oak Ridge Leadership Computing Facility. Contigs smaller than 500 bp were removed. Alignment information was determined by mapping each sample’s reads to the assembly reference with BBtools^15^ (v38.95) [bbmap.sh Xmx450g nodisk = true interleaved = true ambiguous = random mappedonly = t trimreaddescriptions = t usemodulo = t fast = t] to provide an alignment for each sample to the assembly. Overall coverage was determined by running BBTools (v38.95) [pileup.sh] on all alignment files concatenated. A total of 65,176,533,394 reads were input into the aligner and a total of 61,542,936,624 (94%) aligned.

#### MAG generation, refinement, quality check and taxonomic annotation

Assembled contigs were annotated using the DOE-JGI metagenome workflow (v5.1.11)^6^ and grouped into metagenome-assembled genomes (MAGs) using MetaBAT2^7^ (v2.15), an automated metagenome binning software tool that uses an adaptive binning algorithm to eliminate manual parameter tuning. Next, genome completeness and contamination were estimated based on the recovery of a set of core single-copy marker genes using CheckM (v1.1.3)^8^ (Table S1). The bins are reported according to the Minimum Information about a Metagenome-Assembled Genome (MIMAG^16^) standard as high, medium, or low quality. For each of the high- and medium-quality bins, the taxonomic lineage was computed using the GTDB-Tk which is a software toolkit that assigns objective taxonomic classifications to bacterial and archaeal genomes based on the Genome Database Taxonomy (v1.3.0, GTDB database release 95)^9^. The bins identified as low-quality were explored for eukaryotic potential wherein their eukaryotic genome quality (completeness and contamination) and lineage was estimated based on single copy marker gene sets using EukCC (v2.1.2, eukcc2_db_ver_1.2)^17^, and those with more than 50% completion and less than 10% contamination were chosen for further analysis (Table S2). Four of the eukaryotic MAGs were further annotated using JGI’s PhycoCosm annotation pipeline^10^.

#### Viral contig identification, de-replication and taxonomic classification

The computational program geNomad (v1.7.4)^11^ was used to identify viral contigs from unbinned metagenomic data and assign taxonomy. CheckV (v1.5)^12^, was used to determine the completeness and quality of the identified viral sequences (Table S3). Contigs with no completeness estimate, only an hmm-based estimate, only an aai-based low-confidence estimate, and/or a completeness <50% were discarded. Contigs longer than 150% of the aai_expected_length were also removed resulting in a total of 6,350 unique viral sequences.

#### Phylogenomic analysis

NSGTree (v0.4.3; https://github.com/NeLLi-team/nsgtree) was used for phylogenetic tree construction (Fig. 2). The.faa files generated for each MAG and the UNI56.hmm reference set of phylogenetic marker HMMs were used as input files. The Interactive Tree of Life (v6)^18^ was used to visualize and annotate the phylogenetic tree.

## Data Records

The raw shotgun metagenome data has been deposited and is available through NCBI’s SRA and Biosample repository under umbrella project PRJNA1056043 (https://www.ncbi.nlm.nih.gov/bioproject/1056043)^3^, which is organized to include the nested Biosample and SRA Experiment accessions. Table S4 includes all individual metagenomes part of this study with associated GOLD and NCBI biosample and bioproject identifiers and accessions, respectively, and individual resolvable URLs using NCBI’s SRA SRPs. The assembled metagenome has also been made available under PRJNA1134257 (https://www.ncbi.nlm.nih.gov/bioproject/1134257)^4^. Assembled contigs, MAGs, and viral genomes associated with this study are also available under taxon identifier 3300059473 in JGI’s IMG/M platform (https://img.jgi.doe.gov/cgi-bin/m/main.cgi?section=TaxonDetail&page=taxonDetail&taxon_oid=3300059473), along with per-sample alignment files and coverage information available for download on JGI’s Genome Portal (https://genome.jgi.doe.gov/portal/pages/dynamicOrganismDownload.jsf?organism=LakMenMeassembly_FD). High-quality eukaryotic MAGs were further annotated and uploaded onto JGI’s PhycoCosm^10^ as follows: 3300059473_978, https://phycocosm.jgi.doe.gov/Trebou978_1; 3300059473_6682, https://phycocosm.jgi.doe.gov/Treb6682_1; 3300059473_4966, https://phycocosm.jgi.doe.gov/Trebou4966_1; and 3300059473_4402, https://phycocosm.jgi.doe.gov/Trebou4402_1. Associated metadata is available through JGI’s Genomes OnLine (GOLD)^14^ system under GOLD Study ID Gs0136121 (https://gold.jgi.doe.gov/study?id=Gs0136121). Sample metadata and individual metagenome assemblies are available through the National Microbiome Data Collaborative, along with links to the NCBI Biosample identifiers at: https://data.microbiomedata.org/details/study/nmdc:sty-11-5bgrvr62.

## Technical Validation

Technical validation was performed on the metagenome data using established best practices for read quality control, assembly, and annotation. Details of sequencing, read QC, and filtering for each of the 471 individual metagenomes along with software versions and bioinformatics scripts are included in Supplementary Dataset 1. MAG completeness and contamination were assessed using CheckM (v1.1.3) and reported quality was determined according to the MIMAG^16^ standard. For eukaryotic MAGs, estimates for completeness and contamination were assessed using EukCC (v2.1.2). Viral contigs were identified using geNomad (v1.7.4) with completeness and quality of the identified viral sequences assessed using CheckV (v1.5). Evaluation of taxonomic composition of the assembled data was consistent with previous reports of microbial communities recovered from Lake Mendota^2,19^.

## Supplementary information

Supplementary Tables S1-S4

Supplementary Table and Dataset Legends

## Data Availability

The combined assembly used MetaHipMer version 2 with code available here: https://github.com/mgawan/mhm2_staging. Metagenomic analyses used the DOE-JGI Metagenome Annotation Pipeline (v5.1.11)^6^. Detection of viral contigs and quality assessment used geNomad (v1.7.4; https://github.com/apcamargo/genomad) and checkV (v1.5; https://bitbucket.org/berkeleylab/checkv/src/master/). For phylogenetic tree reconstruction, NSGTree (v0.4.3; https://github.com/NeLLi-team/nsgtree) was used.
