# Supplementary material for: Coassembly and binning of a twenty-year metagenomic time-series from Lake Mendota
Source: Sci Data. 2024 Sep 4;11:966. doi: 10.1038/s41597-024-03826-8 (PMC11374980; doi:10.1038/s41597-024-03826-8)
Supplement: Supplementary file 2 — Supplementary Table and Dataset Legends [file 41597_2024_3826_MOESM2_ESM.docx]

**Supplementary Information: Coassembly and binning of a twenty-year metagenomic time-series from Lake Mendota**

**Table S1.** Prokaryotic MAGs recovered with associated quality metrics and taxonomic affiliations.

**Table S2.** Eukaryotic MAGs recovered with associated quality metrics and taxnomic affiliations. PhycoCosm links for reannotated genomes are available for a subset of high-quality MAGs.

**Table S3.** Putative viral contigs identified with associated quality metrics and taxnomic affiliations.

**Table S4.** Individual sample GOLD and NCBI identifiers.

**Dataset 1.** Details of sequencing, read QC, and filtering for each of the 471 individual metagenomes in this study organized by JGI sequencing project identifier. Software versions and bioinformatics scripts are included.
